# Bioassay-Guided Isolation of Anti-Candida Biofilm Compounds From Methanol Extracts of the Aerial Parts of *Salvia officinalis* (Annaba, Algeria)

**DOI:** 10.3389/fphar.2018.01418

**Published:** 2018-12-10

**Authors:** Neila Kerkoub, Sujogya Kumar Panda, Ming-Rong Yang, Jing-Guang Lu, Zhi-Hong Jiang, Hichem Nasri, Walter Luyten

**Affiliations:** ^1^Laboratory of Biodiversity and Pollution of Ecosystems, Department of Biology, University Chadli Bendjedid, El Tarf, Algeria; ^2^Department of Biology, KU Leuven, Leuven, Belgium; ^3^State Key Laboratory of Quality Research in Chinese Medicine, Macau Institute for Applied Research in Medicine and Health, Macau University of Science and Technology, Taipa, China

**Keywords:** sage, carnosol, 12-methoxy-trans-carnosic acid, biofilm, antifungal, *Candida*

## Abstract

*Salvia officinalis* is frequently used in traditional Algerian medicine to treat diverse microbial infections, including oral and vaginal candidiasis. The aerial parts of *S. officinalis* collected in Annaba, Algeria were extracted in parallel by maceration with four solvents viz. hexane, acetone, methanol and water. All the extracts were tested *in vitro* against several *Candida* species: *C. albicans, C. glabrata*, and *C. parapsilosis*. Furthermore, the activity against biofilm-forming *C. albicans* was investigated using bioassay-guided fractionation. A large-scale extract was prepared via maceration in methanol, followed by fractionation on a silica gel column using increasingly polar mixtures of *n*-hexane, ethyl acetate, methanol, and acetic acid as mobile phase, to yield a total of 150 fractions. Two major active fractions (F-31 and F-39), were further separated by HPLC, resulting in several active chromatographic peaks. Carnosol and 12-methoxy-trans-carnosic acid were isolated as two major active compounds, and identified by a combination of NMR and mass spectrometry. The biofilm inhibitory concentration showed that 12-methoxy-trans-carnosic acid is more effective than carnosol with BIC_50_ values of 94 μM (95% confidence interval, 78.9–112.1 μM) and 314 μM (95% confidence interval, 200.7–491.2 μM), respectively. The present study supports the traditional use of sage in the treatment of various fungal infections caused by *Candida*. Further studies of the bioactive compounds in an *in vivo Candida* biofilm model are required to validate their clinical potential as antifungals.

## Introduction

Over the past decades, the incidence of fungal infections has dramatically increased, especially for systemic ones, due to a combination of reasons: the growing use of invasive medical devices (e.g., intravascular and urinary catheters, as well as implanted prostheses), and the increased use of broad-spectrum antimicrobial as well as immunosuppressive therapies (Kojic and Darouiche, 2004; Pfaller et al., 2014; Nett and Andes, 2015). Moreover, there is an increasing number of patients who survive with predisposing—(e.g., diabetes) or debilitating diseases, or a compromised immune system (Garber, 2001; Pilmis et al., 2016). The genus *Candida* counts more than 200 species, of which ~10% are human pathogens (Manzoor et al., 2016). These are able to cause infection that range from mild cutaneous—or mucosal—to severe systemic infections. Among *Candida* species, *Candida albicans* remains the most frequently isolated fungus from hospitalized patients (Lohse et al., 2018). However, an increase in the proportion of infections caused by non-*albicans Candida* has been observed recently (Pfaller et al., 2014; Quindos, 2014; Gong et al., 2016). Despite advances in antifungal therapy, *Candida* infections continue to have a major impact on mortality and morbidity, as well as on the duration and cost of hospitalization (Tanwar et al., 2014). This situation has led pharmaceutical companies and researchers to explore new alternatives, in order to discover improved antifungal agents that satisfy efficacy, safety, and economic criteria. Medicinal plants constitute one of these alternatives. “Increasing trends of health organizations and pharmaceutical industries to use plants as safe and effective alternative sources of synthetic antifungals are due to major problems of slow growing and high costs of synthetic pharmaceutics, their life-threatening side effects, rapid increase in new fungal infections, and dramatic emergence of multidrug-resistant fungal pathogens.” (Razzaghi-Abyaneh and Rai, 2013; Razzaghi-Abyaneh et al., 2013).

*Salvia officinalis* L. (family Lamiaceae), is the most common species of the genus, widely growing throughout the Mediterranean and Middle East (Giannouli and Kintzios, 2000). *Salvia* (sage) is a perennial round shrub; its leaves and flowering tops are aromatic and used for the production of essential oils. Since antiquity this plant has been recognized for its medicinal importance (Ghorbani and Esmaeilizadeh, 2017). It is used for the relief of pain, for protecting the body against oxidative stress, free radical damage, angiogenesis, inflammation, bacterial, and viral infections, as well as for a range of diseases including those of the nervous system, of heart and blood circulation, of the respiratory, digestive, metabolic, and endocrine system, etc. (Istudor, 2001; Hamidpour et al., 2014). Algeria is one of the Maghreb countries where phytotherapy is frequently used by the population (Allali et al., 2008). Based on recent publications of ethnopharmacology surveys in Algeria, we found that this plant is most commonly used, with use-values of 0.48 (Boudjelal et al., 2013), 1.875 (Ouelbani et al., 2016), and 2.29 (Telli et al., 2016). In fact, sage is used in the Ouargla province (South-Eastern Algeria) to treat foot ulcers, which are common there (Telli et al., 2016). In the Msila region (North Algeria), the infusion from its aerial parts is mainly used for diabetes, weight loss, as antihypertensive and for eczema (Boudjelal et al., 2013). Similarly, people living in the region of Constantine and Mila (North-East Algeria) recommend it for the treatment of diverse diseases including nervous system diseases, muscle pain, headache, memory problems, as analgesic, as antispasmodic, for wounds, influenza, rheumatism, cholesterol lowering, gynecological diseases, and teeth problems (Ouelbani et al., 2016). Another recent survey conducted in the region of Annaba and El Tarf (extreme North-East, Algeria) revealed the use of *S. officinalis* alone or in combination with other medicinal plants to treat and relieve oral and vaginal yeast infections (Kerkoub, unpublished observations).

A decade ago, Horiuchi et al. (2007a) isolated from *Salvia* ursolic and oleanolic acid, which possess antibacterial activity. Although *Salvia* is commonly used for several infections in Algeria, as well as in other parts of the world, the compounds responsible for its antifungal properties remain not well studied (or fully explored). Therefore, the aim of the present study is to isolate the anti-Candida biofilm constituents from *Salvia officinalis* aerial parts through bioassay-guided purification.

## Materials and Methods

### Chemicals and Reagents

Acetone, acetonitrile, methanol, hexane, and ethyl acetate (all of HPLC grade) were purchased from Sigma-Aldrich Co. (USA). Sterile deionized water was produced by a water purification system (Milli-Q Reagent Water System, MA, USA). Yeast extract and Bacto™ peptone were purchased from Lab M Ltd. (Lancashire, UK) (Panda et al., 2018). Dimethyl sulfoxide (DMSO, molecular biology grade), dextrose, amphotericin B, (±)-miconazole nitrate salt, Roswell Park Memorial Institute (RPMI) medium with L-glutamine without sodium bicarbonate, and 4-morpholinepropanesulfonic acid (MOPS) were purchased from Sigma-Aldrich (St. Louis, MO, USA). Resazurin dye was purchased from Acros Organics (New Jersey, USA) (Kipanga and Luyten, 2017).

### Collection and Identification of Plant

**Figure 1:**
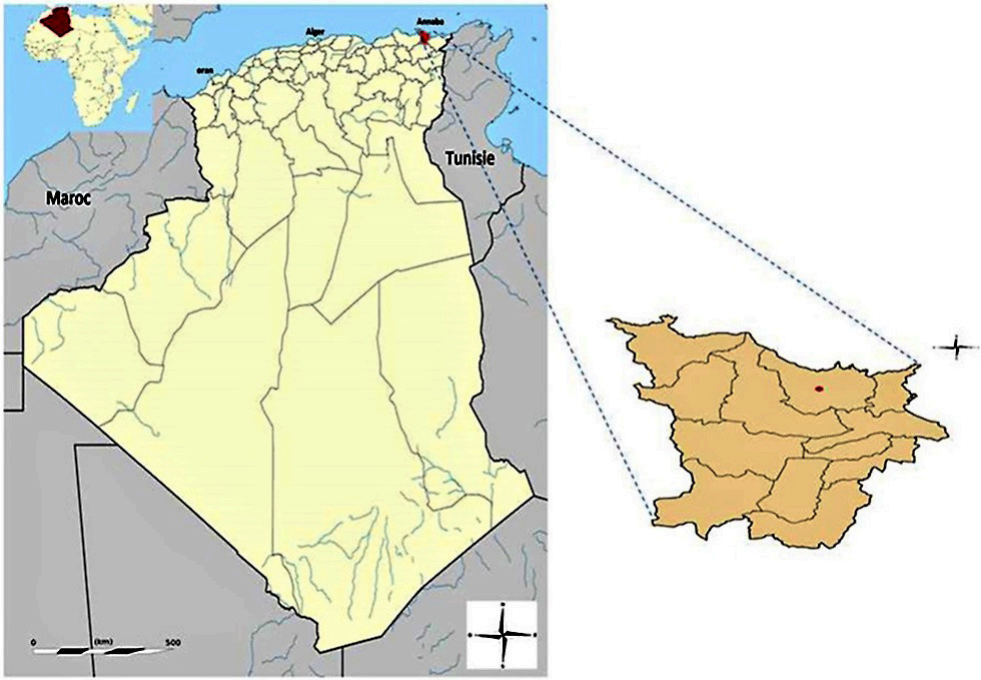
Map showing the zone of collection of *Salvia officinalis*.

The flowering aerial parts of *Salvia officinalis* L. were collected in May 2014 from Annaba, situated in the Northeast of Algeria, at a latitude of 36°54'38.78”N and longitude of 7°41'08.96”E (Figure 1). The plant material was identified by an experienced botanist (Lakehal Samia) using the descriptive reference of (Quezel and Santa, 1963). A voucher specimen (code 05_14) was preserved and stored in the herbarium of the Department of Biology, University Chadli Bendjedid, El Tarf, Algeria.

### Extraction of Plant Materials

The collected plant parts were dried for 1 week at ambient temperature to maintain their green color and volatile oils (Panda, 2014). The dried raw botanical material was ground to a fine powder. Small-scale extractions were performed as described in our previous study (Panda et al., 2018). In short, 1 gram of powder was transferred separately into each of four 15 mL sterile Falcon tubes, and extracted with four different solvents: acetone, hexane, methanol and water. One mL aliquots of each extract were dried by evaporation of the solvent, and the dried residue was re-dissolved in DMSO (for the organic solvent extracts), or in water (for the aqueous extract) in order to prepare a stock solution of 50 mg/mL. All the samples were stored at 4°C until further testing.

### Large-Scale Extraction

Approximately 250 g of the dried plant was weighed and transferred to a 2.5 L glass bottle; 1 L methanol was added and vigorously mixed. The bottle was placed in a sonication water bath for four times 30 min every 6 h to maximize extraction. Then, the extract was filtered (185 mm, MACHEREY-NAGEL, Germany) and the filtrate dried on a rotary evaporator (BUCHI rotavapor R-100). The extraction process was repeated until the yield became negligible.

### Bioassay-Guided Fractionation and Isolation

The dried residue of the methanol extract (21.7 g) was adsorbed to silica gel (Sigma-Aldrich, high-purity grade, pore size 60 Å, 70–230 mesh) and separated on a cylindrical glass column (600 mm height × 55 mm diameter). The elution was performed at the flow rate of 40 mL/min with a step-gradient (Waters, model 600) starting from hexane:ethyl acetate (10:0, 7.5:2.5, 5:5, 2.5:7.5) followed by mixture of ethyl acetate:methanol (10:0, 9.5:0.5, 9:1, 8.5:1.5, 8:2, 6:4, 4:6, 0:10), then methanol:acetic acid (9.5:0.5, 9:1, 8.5:1.5). The eluate was monitored by an absorbance detector model 2487 Dual λ at 360 and 254 nm. In each step of the gradient, 10 fractions of 40 mL each were collected in 50 mL conical Falcon tubes with screw caps. One mL aliquots of each fraction were dried, and the residue dissolved in 60 μL of DMSO. Four μL of this solution was used for bioactivity testing.

### Fungal Strains and Culture Condition

The following *Candida* strains were used for antifungal tests: *C. albicans* strains IHEM3732 and SC5314 (the latter for biofilm), *C. glabrata* ATCC 2001 and *C. parapsilosis* ATCC 22019. All strains were stored in yeast-extract peptone dextrose (YPD) broth and 20% glycerol at −80°C in cryovials. Material from the frozen stock was streaked out on YPD agar plates, which were incubated overnight at 37°C (Kipanga and Luyten, 2017).

### Activity Against Planktonic *Candida* Species

A microdilution broth method was used as described previously by Panda et al. (2017) to study the anti-Candida activity in planktonic growth. A standardized inoculum was obtained by growing the test organisms overnight and diluting that starter culture to a turbidity of OD = 0.003 at 620 nm. For antifungal activity, each well of a microtitre plate was inoculated with 196 μL of the diluted yeast suspension, and 4 μL of the extract (50 mg/mL stock solution) or a dilution of it was added (for a final concentration starting at 1,000 μg/mL). Control wells were prepared with 196 μL YPD broth and 4 μL extract to correct for any absorption due to extract components. Control wells were filled with 196 μL YPD broth and 4 μL of DMSO or MilliQ, or the antifungal amphotericin B (at 20 μg/mL in DMSO). The percent inhibition of *Candida* strains was calculated according the following formula:

$$\begin{array}{l} Inhibition\;\left(\%\right)=100-\left(\frac{A-B}{C}\times 100\right) \end{array}$$

Where A is the OD value of a well with microbial culture and test sample, B the OD value of the corresponding negative control well with a mixture of pure broth and test sample and C the OD value of the average of two or more solvent control wells.

### Biofilm Formation and Determination of Biofilm Inhibitory Concentration (BIC_50_)

The anti-biofilm activity of fractions and isolated compounds was determined by following the method described recently by Kipanga and Luyten (2017) on *C. albicans* SC 5314. An overnight culture of *C. albicans* in YPD media was centrifuged at 800 rpm for 2 min and the supernatant was discarded. The pellet was then washed with RPMI medium. A standardized inoculum was obtained by adjusting OD = 0.1 at 600 nm (about 10^6^ cells/mL). Then, 100 μL of this suspension was distributed in 96-well flat-bottom plate and incubated in a stationary incubator for 90 min at 37°C to permit adhesion. Afterwards, each well was carefully washed using PBS without disturbing the biofilm layer at the bottom. A two–fold serial dilution (up to 32–fold) of a test compound was prepared in a 96–well conical-bottom (V) polystyrene microtitre plate. Four μL of the test compound was added gently with 196 μL fresh RPMI-MOPS medium. The plates were then incubated at 37°C in a stationary incubator. Following 24 h of incubation, the medium was carefully aspirated and the cells washed once with PBS and stained with 100 μL resazurin dye (0.4% v/v) in the absence of light. After 1 h of incubation at 37°C, fluorescence was measured with λ_ex_ at 535 nm and λ_em_ at 590 nm using a FLEXStation II (Molecular Devices). Control wells were filled with 196 μL medium and 4 μL of DMSO or the antifungal amphotericin B (at 20 μg/mL in DMSO). The percent inhibition of *Candida* strains was calculated according the following formula:

$$\begin{array}{l} Biofilm\;inhibition\;\left(\%\right)=100-\left(\frac{A-B}{C}\times 100\right) \end{array}$$

Where A is the fluorescence readings of (biofilm & antimicrobial), B is fluorescence readings of well without biofilm and C is the average of three solvent controls (DMSO).

Data from dose-response experiments were represented as the percent of inhibition compared to control, and analyzed by non-linear regression with Prism™ (GraphPad Prism 5.0 Software Inc., San Diego, CA). The BIC_50_ for each growth condition was calculated by fitting the data to a non-linear least-squares sigmoid regression curve, fixing the top and bottom of the curve at 100 and 0 percent, respectively. The BIC_50_ corresponds to the concentration that would yield an inhibition of 50%; the nonlinear regression algorithm also estimates a 95% confidence interval for the BIC_50_.

### HPLC-DAD Analysis

HPLC analysis was performed on a Shimadzu, LC-20AT system (model DGU 20A3) equipped with LC-20AT quaternary pump, a DGU-20A3/DGU-20A5 on-line degasser, a SPD-20A photodiode array detector, and a CBM-20A/20A interface. The chromatography data were acquired and processed using Lab Solution software. Plant samples were filtered through a CHROMAFIL^®;^ Xtra H-PTFI filter (pore size 0.45 μm, filter 13 mm, MACHEREY-NAGEL, Germany) prior to HPLC injection. The extracts were analyzed using a reverse-phase HPLC column: Sunfire™ prep C18 column (10 × 250 mm, 5 μm) (Waters, Ireland). The mobile phase was composed of solvent A (H_2_O with 0.1% trifluoro acetic acid, Acros Organics) and solvent B (acetonitrile (ACN) with 0.1% TFA). A flow rate of 4.0 mL/min was used at 20°C. After the HPLC condition was optimized, the gradient used for fraction 31 (F31) was: starting with the 65% ACN (in water) for 5 min, and linearly increasing the ACN from 65 to 70% over 5–7 min, and from 70 to 75% over 7–27 min, and from 75 to 80% over 27–47 min, and from 80 to 100% over 47–55 min, followed by eluting with 100% ACN for 5 min. Similarly, for fraction 39 (F39) the gradient was: starting with the 60% ACN (in water) for 5 min, and linearly increasing the ACN from 60 to 100% over 5–40 min, followed by eluting with 100% ACN for 5 min. The fractions were collected every minute and dried in a SpeedVac Concentrator, then dissolved in 12 μL DMSO each, and tested for anti-Candida activity. The active fractions were linked to the corresponding peaks by aligning the activity profile with the corresponding chromatogram (Liu et al., 2018).

### UHPLC-QTOF MS Analysis

LC-MS analyses of isolated compounds were performed on an Agilent 1290 Infinity UHPLC system (UHPLC, Agilent Technologies, Santa Clara, CA, USA) coupled with a Bruker maXis impact mass spectrometer (QTOF, Bruker, Switzerland). Electrospray ionization (ESI) mass spectra were acquired in positive and negative ion mode. The results were recorded with the following ESI source parameters: end plate offset voltage of 500 V, capillary voltage of 4,000 V, nebulizer of 2.5 bar and dry gas flow of 8.0 L/min at 200°C. The chromatographic separation was performed on an Agilent poroshell 120 EC-C18 column (150 mm × 3.0 mm, 2.7 μm) with a constant temperature of 30°C. The flow rate was 0.35 mL/min and the injection volume were 5 μL with a concentration of 1 μg/mL. The mobile phase consisted of 0.1% formic acid in water (A) and 0.1% formic acid in acetonitrile (B). A linear gradient was optimized as follows: 0–8 min, 5–95% B; 8–10 min, 95% B; 10–11 min, 95–5% B, and finally equilibration with 5% B for 3 min (Liu et al., 2018).

### NMR Spectroscopy

All experiments were performed on a Bruker Ascend LH 600 MHz NMR spectrometer (Bruker, Switzerland) operating at NMR frequency of 600 MHz for ^1^H and 150 MHz for ^13^C NMR. It was equipped with a 5 mm CryoProbe (CP DCH 600S3 C/H-D-05 Z) in deuterated methanol solution at 298°K. All ^1^H (600 MHz) and ^13^C NMR (150 MHz) spectra were recorded with chemical shifts in δ (ppm) and coupling constants (J) in hertz (Hz).

## Results

**Figure 2:**
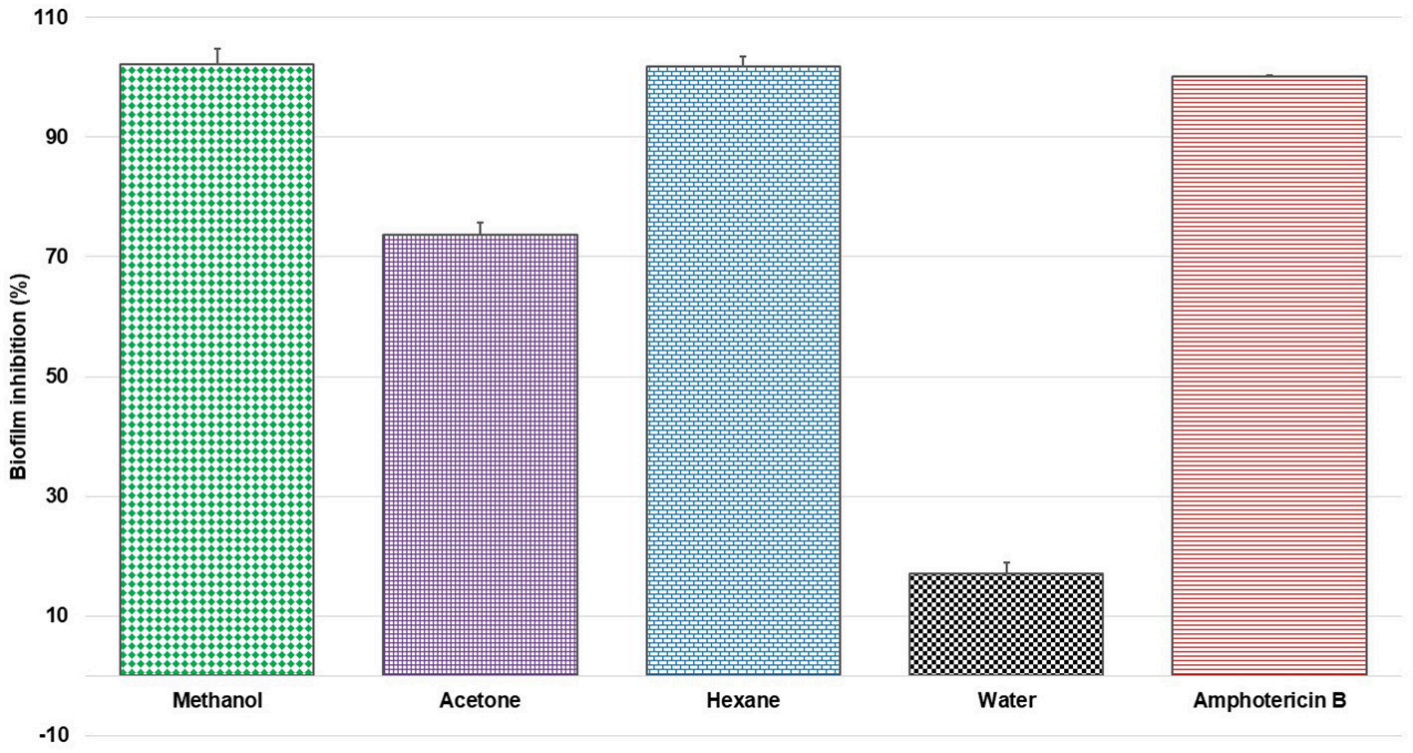
Percent inhibition of *C. albicans* biofilm with extracts of *S. officinalis* (1 mg/mL) prepared in different solvents (mean ± SD); positive control amphotericin B.

**Figure 3:**
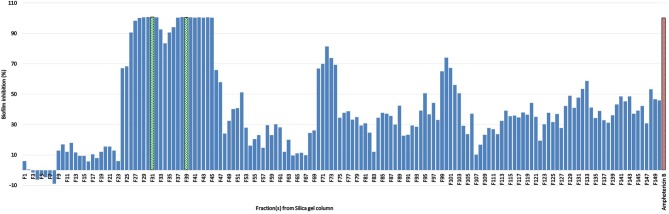
Percent inhibition of *C. albicans* biofilm treated with one of 150 fractions collected from silica gel column chromatography of *S. officinalis* methanol extract; positive control amphotericin B; fraction 31 (F 31) and 39 (F 39) in green shows the strongest activity (see Figure 4).

**Figure 4:**
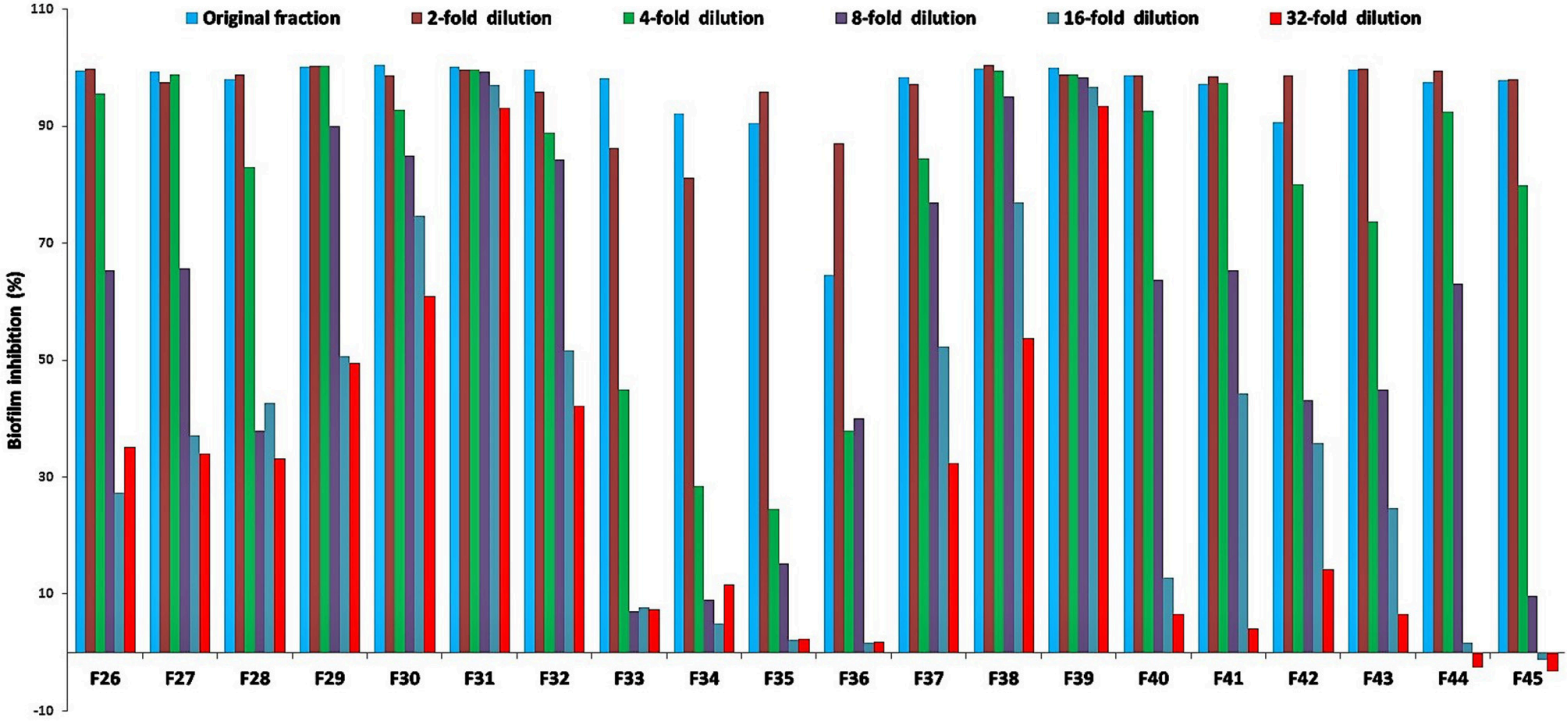
Percent inhibition of *C. albicans* biofilm treated with two-fold serial dilutions of select active fractions (BIC_50_ in mg/mL for fraction F26 = 800, F27 = 5,700, F28 = 1,900, F29 = 1,860, F30 = 1,500, F31 = 340, F32 = 520, F33 = 600, F34 = 800, F35 = 780, F36 = 550, F37 = 660, F38 = 1,450, F39 = 530, F40 = 480, F41 = 450, F42 = 100, F43 = 560, F44 = 540, F45 = 560, starting concentration in μg/mL) from silica gel column; positive control amphotericin B; fractions 31 and 39 show the strongest inhibition, and were selected for further analyses (Figures 5 and 6).

For the initial testing, four different solvents viz. acetone, hexane, methanol, and water were used to extract the aerial parts of *S. officinalis*, and the yield and appearance are shown in Table 1. All these extracts were tested for antifungal activity (*in vitro*) against different *Candida* species (Table 2). The methanol extract was most effective against both biofilm and liquid culture of *C. albicans* (Figure 2). Therefore, a large-scale methanol extract was prepared from 250 g plant material, yielding 21.7 gram of dry extract. This was separated on a silica gel column with a hexane-ethyl acetate-methanol-acetic acid step-gradient, resulting in 150 fractions. Several of these (Figure 3) were effective against *C. albicans* biofilm. Most of the activity was found in fractions between 26 and 45 (with apparently two regions of maximum activity: F26 to 33 and F37 to 45); minor activity was centered around F72 and F100. Twenty fractions (F26 to 45) were further tested by two-fold serial dilution to determine the most active fraction (Figure 4). Fraction 31 and 39 were the most potent, and were further separated by HPLC.

**Table 1:** Yield, color, and physical appearance of extracts.

**Type of extract**	**Yield in mg dried extract per gram starting material**	**Color, consistency of dried extract**
Hexane	130	Light green, hard
Acetone	100	Green, sticky
Methanol	90	Dark green, sticky
Water	260	Reddish brown, sticky

**Table 2:** Activity (% inhibition of growth) against planktonic *Candida* cells of small-scale extracts in various solvents of *S. officinalis*.

***Candia* species**	**Hexane**	**Acetone**	**Methanol**	**Water**
*Candida albicans*	68 ± 1.9	52 ± 0.7	98 ± 1	96 ± 3.6
*Candida parapsilosis*	98 ± 0.4	94 ± 0.4	81 ± 1	79 ± 0.6
*Candida glabrata*	76 ± 1	43 ± 3	34 ± 0.4	100 ± 0.6

*Concentration of extract; 1 mg/mL; % inhibition mean ± SD*.

**Figure 5:**
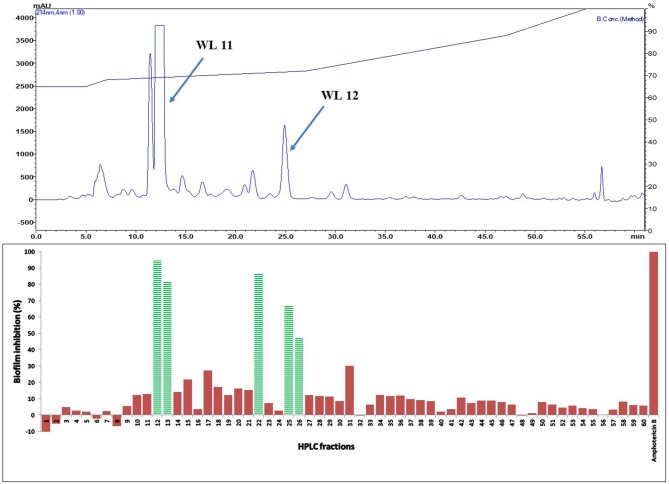
**(Top)** HPLC chromatogram of fraction 31 (F 31) of silica gel column (see Figure 3); fractions were collected per minute and tested for activity (percent inhibition of *C. albicans* biofilm) (**Bottom**); positive control amphotericin B; compounds WL11 and WL12 in the most active peaks were analyzed by mass spectrometry and NMR (see Table 3).

**Figure 6:**
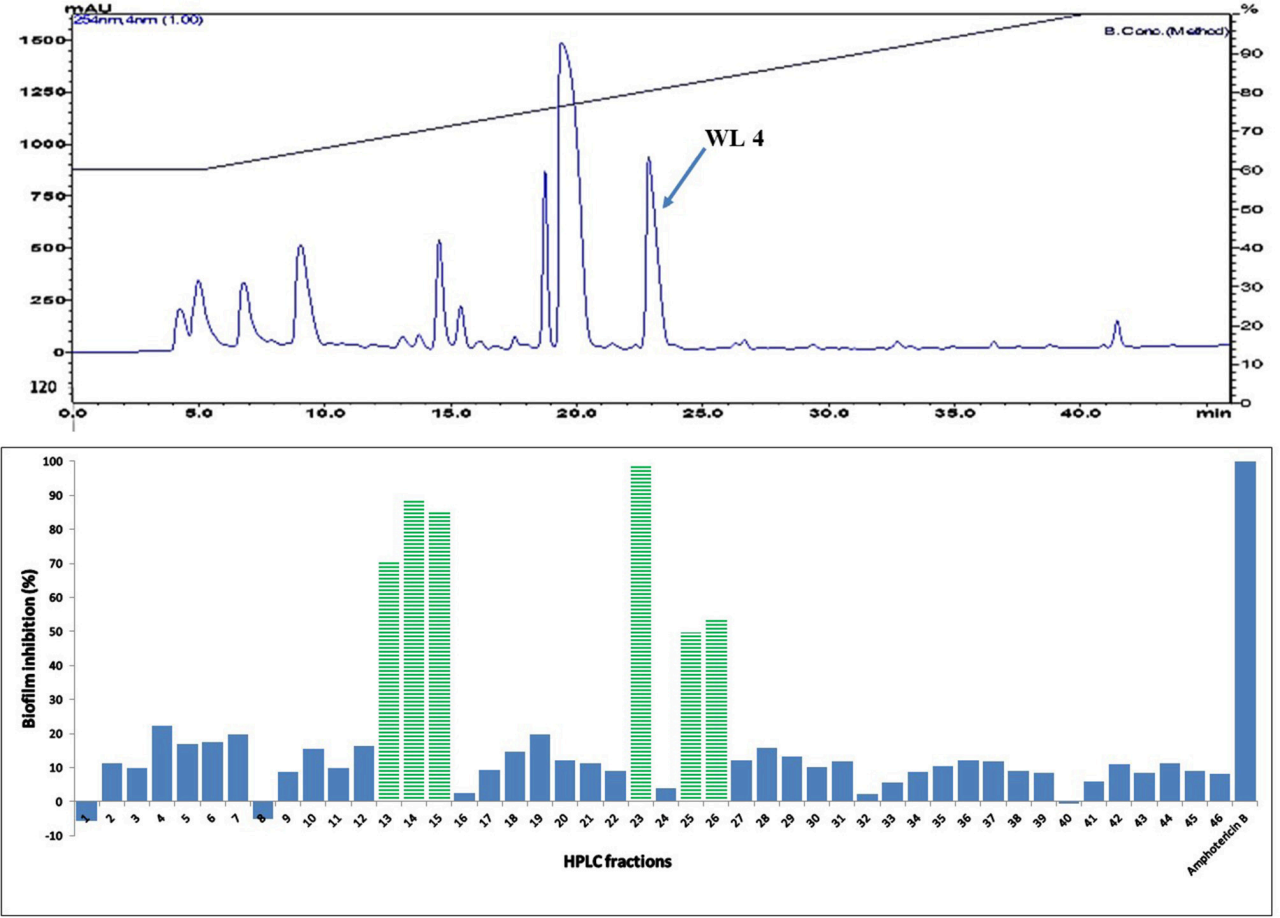
**(Top)** HPLC chromatogram of fraction 39 (F 39) of silica gel column (see Figure 3); fractions were collected per minute and tested for activity (percent inhibition of *C. albicans* biofilm) **(Bottom)**; positive control amphotericin B; compound WL4 in the most active peak was analyzed by mass spectrometry and NMR (see Table 3).

Active fractions 31 and 39 were dried and re-dissolved in 65 and 60 % acetonitrile-water, respectively. Both samples were injected on a SunFire™ C18 semi-preparative column (Waters) and separated by an acetonitrile gradient as described in the section on HPLC-DAD Analysis. All the sub-fractions of F31 and F39 were collected, dried and dissolved in DMSO (12 μL), and tested for activity against *C. albicans* in biofilm assays (Figures 5 and 6, respectively).

**Figure 7:**
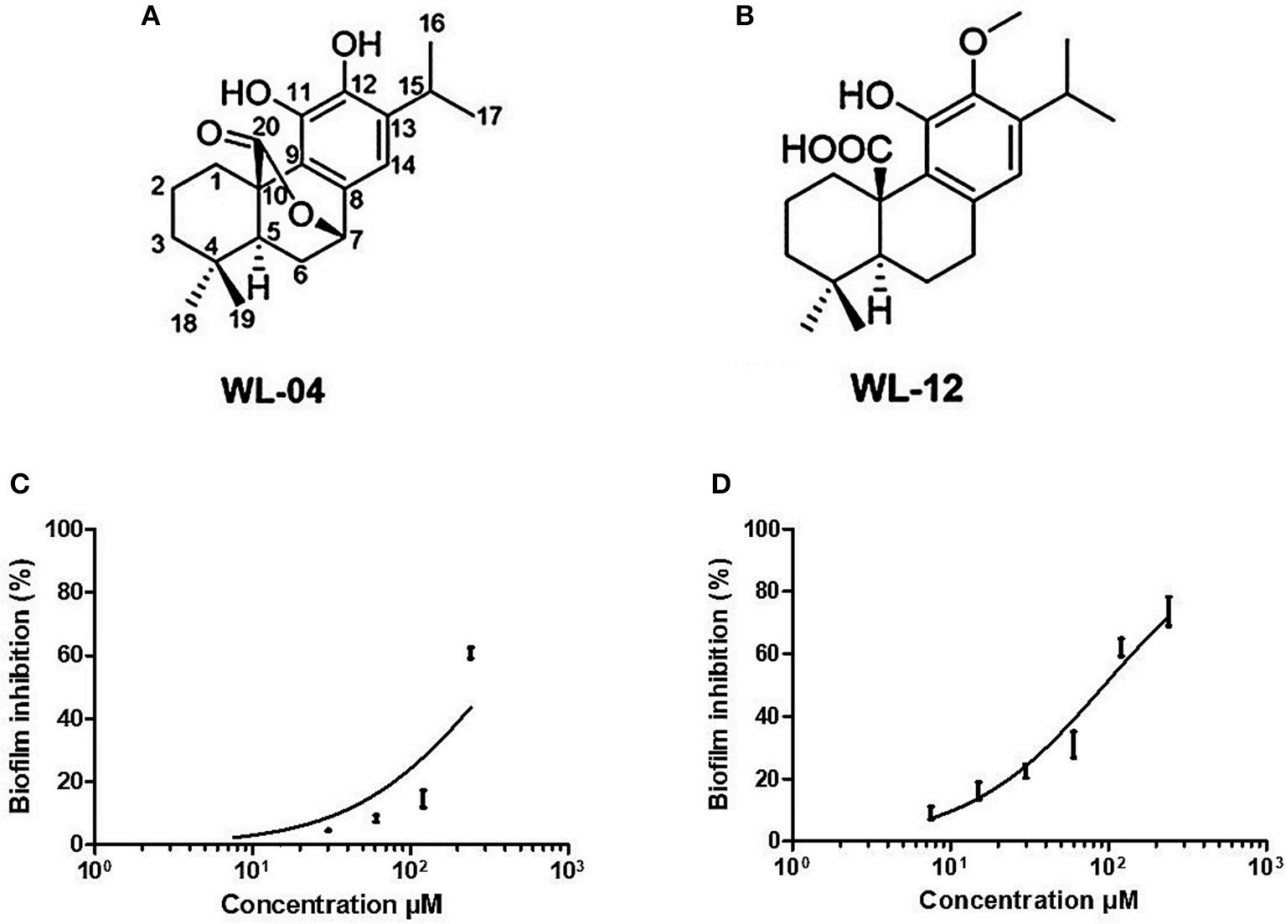
Structures of carnosol **(A)** and 12-methoxy-trans-carnosic acid **(B)**, isolated from the aerial parts of *S. officinalis*; dose-response curve of carnosol **(C)** and 12-methoxy-trans-carnosic acid **(D)**. Data from dose-response experiments were represented as the percent of inhibition, and analyzed with GraphPad Prism 6 software (San Diego, CA, USA). A non-linear regression of log(inhibitor) vs. response, with bottom constrained to 0 and top constrained to 100, and Hill slope equal to 1, was used to estimate the BIC_50_ including its 95% confidence interval.

The two purified active peaks eluting at 25 and 23 min from F31 and F39, respectively, were further analyzed by NMR and LC-MS, and in each a single compound was identified (WL12 and WL04) (Supplementary Material). A summary of their ^1^H-NMR and ^13^C-NMR spectra is presented in Table 3. WL04 (peak eluting at 23 min from fraction 39), was identified as carnosol (molecular formula C_20_H_26_O_4_; Figure 7A) based on positive-ion HRESIMS m/z 331.1908 [M+H]^+^ (calcd. 331.1904 for C_20_H_26_O_4_) and negative-ion HRESIMS m/z 329.1768 [M-H]^−^ (calcd. 329.1758 for C_20_H_26_O_4_). Its BIC_50_ was estimated from dose-response experiments as 104 μg/mL (314 μM, 95% confidence interval, 200.7 to 491.2 μM) (Figure 7C). WL12 (peak at 25 min of fraction 31) was identified as 12-methoxy-trans-carnosic acid (molecular formula C_21_H_30_O_4_; Figure 7B) based on positive-ion HRESIMS m/z 347.2218 [M+H]^+^ (calcd. 347.2217 for C_21_H_30_O_4_) and negative-ion HRESIMS m/z 345.2083 [M-H]^−^ (calcd. 345.2071 for C_21_H_30_O_4_). The BIC_50_ of WL12 is 31 μg/mL (94 μM, 95% confidence interval, 78.9–112.1 μM) (Figure 7D). The tentative identifications by mass spectrometry were confirmed by NMR. Another active peak (WL11) was isolated from fraction 31 eluting at 14 min but could not be identified based on LC-MS and NMR because of impurities. However, according to ^13^C-NMR, it is probably a sesquiterpene (Supplementary Material).

**Table 3:** ^1^H-NMR (600 MHz) and ^13^C-NMR (150 MHz) spectral data of WL04 and WL12 in CD_3_OD.

**Position**	**Compound WL04**		**Compound WL12**	
	**^**1**^H (*J* in Hz)**	**^**13**^C**	**^**1**^H (*J* in Hz)**	**^**13**^C**
1	2.57 ddd (13.9,13.9,4.2)	30.1	2.81 overlapped	35.4
	2.81 brd (13.9)		3.66 overlapped	
2	1.60 m	20.0	1.53 m	20.6
	1.92 m			
3	1.33 ddd (13.5,13.5,3.4)	42.2	1.10 ddd (13.5,4.5)	42.6
	1.52 brd (13.5)		1.32 ddd (13.5,4.5)	
4		35.5		34.9
5	1.70 dd (10.7,5.8)	47.0	1.51 overlapped	55.6
6	1.86 ddd (13.9,10.7,1.6)	30.9	1.81 m	19.7
	2.20 ddd (13.9,5.8,4.2)		2.26 m	
7	5.42 dd (4.2,1.6)	79.7	2.32 overlapped	33.2
			2.80 overlapped	
8		133.3		135.3
9		123.0		128.4
10		49.8		34.9
11		144.2		149.7
12		144.7		144.2
13		136.0		140.6
14	6.70 s	112.5	6.47 s	118.4
15	3.25 m	27.9	3.18 m	27.5
16	1.19 d (7.2)	23.20	1.18 d (7.0)	24.0
17	1.20 d (7.2)	23.23	1.19 d (7.0)	24.1
18	0.876 s	32.2	0.98 s	33.2
19	0.872 s	20.1	0.92 s	21.2
20		179.4		180.0
OMe			3.66 s	61.7

## Discussion

### Antifungal Activity of *S. officinalis*

The *in vitro* antimicrobial activity of *Salvia officinalis* was demonstrated decades ago (Jalsenjak et al., 1987), and was attributed to the presence of 1,8-cineole, thujone and camphor (Jalsenjak et al., 1987; Sur et al., 1991). Indeed, *S. officinalis* essential oil characteristically contains high concentrations of 1,8-cineole, thujone, and camphor. Essential oils of *Salvia officinalis* have been studied for activity against many *Candida* species (*C. albicans, C. parapsilosis, C. krusei*, and *C. tropicalis*), in addition to filamentous fungi, such as *Aspergillus* species and dermatophytes (Khalil and Li, 2011; Abu-Darwish et al., 2013; Sookto et al., 2013; Rus et al., 2015). Pinto et al. (2007) studied the antifungal activity of essential oils of *Salvia officinalis* and found potent activity (1.25-10 μL/mL) against several *Candida* species, including *C. albicans*. Also, Sookto et al. (2013) found that *S. officinalis* essential oil exhibits activity against different strains of *C. albicans*. It appears that most previous studies on the antimicrobial (and specifically the antifungal) activity of *S. officinalis* have focused on its essential oil. Our study, on the other hand pertains to extracts containing primarily non-volatile components. Differences between the anti-Candida effects of *Salvia officinalis* extracts and essential oils may be due to the much higher concentration of compounds in, as well as different composition of the latter. For instance, the MIC of 1,8-cineole for *C. albicans* is 32 mg/mL (Simşek and Duman, 2017), for thujone 60 μg/mL and for camphor 125 μg/mL (Edris et al., 2007).

### Antifungal Activity of Methanol Extract of *S. officinalis*

Cardoso et al. (2012) tested a 100 mg/mL concentration of *S. officinalis* tincture and did not find activity against *C. albicans* and *C. tropicalis*. Similar observations were also reported by Tan et al. (2016) for an ethanol extract of the aerial parts of *S. officinalis* (MIC >400 μg/mL against *C. albicans*), while the essential oil showed very potent activity (MIC 3.2 μg/mL). Martins et al. (2015) found that methanol extracts of *S. officinalis* have activity against two *C. parapsilosis* strains (out of five) and one *C. tropicalis* strain (out of five), whereas no activity against four *C. albicans* strains was observed. It is not clear why alcoholic extracts were previously reported repeatedly to show no anti-Candida activity, whereas we could demonstrate it easily with methanol extracts. Perhaps it is due to differences in the location and time of collection of the botanical material, as well as post-harvest processing or extracting. In any case, our report seems to be the first to document anti-Candida activity in methanol extracts of *S. officinalis*. Methanol is also believed to be a suitable solvent as it can efficiently penetrate cell membranes, permitting extraction of higher levels of endo-cellular components than solvents with lower polarity, and shows better antimicrobial properties (Silva et al., 1998; Panda, 2014; Panda et al., 2016).

### Antifungal Compound From *S. officinalis* Extracts

Several studies on plant extracts from this species have focused on antimicrobial properties (Bozin et al., 2007; Delamare et al., 2007; Horiuchi et al., 2007a,b; Jasim and Al-khaliq, 2011; Velickovic et al., 2011; Cardoso et al., 2012) but have not identified the bioactive compounds. Recently, Martins et al. (2015) studied phenolic compounds and their antioxidant and antimicrobial activities against medical isolates of *Candida* in aqueous (prepared by infusion and decoction) and methanol/water (80:20, v/v) extracts from *Salvia officinalis*. The major compounds identified by LC-MS were derivatives of caffeic acid, rosmarinic acid, salvianolic acid, sagerinic acid, and luteolin, but the relationship between the presence of these compounds and the antifungal activity was not established (Martins et al., 2015). Therefore, the compounds responsible for the antifungal activity of (methanol) extracts of *S. officinalis* have not been established so far, and our identification of carnosol and 12-Methoxy-trans-carnosic acid by bioassay-guided purification is therefore the first identification of two compounds underlying the antifungal activity of solvent extracts from this plant. Previous studies probably missed them because they are not major compounds identified in typical phytochemical studies.

### Isolation of Carnosol and 12-Methoxy-trans-carnosic Acid From *Salvia* Species

Carnosol was initially isolated from *Salvia carnosa* (White and Jenkins, 1942a,b) and is presumably identical with a compound obtained from *Salvia officinalis* (Janot et al., 1952). Horiuchi et al. (2007b) found that carnosol from *Salvia officinalis* has weak antimicrobial activity. Both carnosol and 12-methoxy-trans-carnosic acid were reported as major components of *Salvia fruticosa* (Exarchou et al., 2014; Scheler et al., 2016). The compounds were also reported in *S. officinalis* (Fischedick et al., 2013), but not as major constituents. This probably explains why they have not been identified so far as responsible for the antifungal activity of *S. officinalis*.

### Antifungal Activity of Carnosol and Its Analogs

Abietane diterpenes (like carnosic acid) are a promising chemical class due to their abundance in medicinal plants and occurrence in industrial wastes (San Feliciano et al., 1993). A recent review by González (2014) on aromatic abietane diterpenoids and their biological activities devoted special attention to their antimicrobial activity. The compounds were demonstrated to have antioxidant (Richheimer et al., 1996), antibacterial (Oluwatuyi et al., 2004), antifungal (Gigante et al., 2003; Exarchou et al., 2014), and cytotoxic activities (Aoyagi et al., 2006).

Fischedick et al. (2013), studied the structure-activity relationship of phenolic diterpenes from *Salvia officinalis* as activators of the nuclear factor E2-related factor 2 pathway. From *Salvia officinalis*, these authors isolated carnosol, epirosmanol, rosmanol, carnosaldehyde, carnosic acid, 12-methoxy-carnosic acid and sageone using different chromatographic techniques (Fischedick et al., 2013). Dimayuga et al. (1991) also studied the antimicrobial activity of carnosol from *Lepechinia hastata* (*Lamiaceae*) against a wide range of microorganisms, and concluded that this compound inhibits *Staphylococcus aureus, Bacillus subtilis, Escherichia coli*, and *Candida albicans* (~0.4 mg per disc). Later, Dimayuga again studied the activity of carnosol in more pure form against the same strains, and concluded that the compound was active only against Gram-positive but not against Gram-negative bacteria, nor against the yeast *C. albicans*. They explained that the probable reason for their different results was the impure nature of the previous isolates, which had activity against many more test pathogens.

The diterpene carnosic acid 12-methyl ether (12-methoxycarnosic acid) was also isolated from *S. microphylla* as a major antimicrobial constituent, but was reported not active against *C. albicans* (Aydogmuş et al., 2006). Jordán et al. (2012) studied how the relative amounts of carnosol, carnosic acid, and rosmarinic acid from methanol extracts of *Rosmarinus officinalis* affect the antioxidant and antimicrobial activities. These authors conclude that the antibacterial efficacy improved when carnosol was the major diterpene component. The antifungal activity of carnosol and 12-methoxy-carnosic acid has so far been reported as weak. It is not clear why our findings are different, but the fact that some of the previously reported carnosol preparations were not pure, complicates the interpretation of their bioactivity. Further mechanism of action studies and molecular modeling need to be performed to identify the molecular targets of these compounds (De Monte et al., 2015, 2016).

### Synergistic Effects of Carnosol

Fischedick et al. (2013) report that the activity of carnosic acid, carnosol, and hispidulin are in the same concentration range as the extract itself, suggesting that the bioactivity cannot be solely attributed to a single constituent, and that synergies may be at work. Horiuchi et al. (2007b) studied synergy of carnosol from *Salvia officinalis* in combination with several antibiotics such as arbekacin, gentamicin, streptomycin, ethidium bromide, erythromycin and tetracycline; carnosol greatly reduced the MIC of gentamicin. Most researchers have found that carnosol has weak antimicrobial activity, but greatly reduced the MICs of various aminoglycosides and other types of antimicrobial agents (Horiuchi et al., 2007b). This may be due to efflux pump modulation by dissipation of the bacterial membrane potential (Ojeda-Sana et al., 2013). It is not clear whether carnosol can also synergize with clinical antifungals. However, comparing our antifungal activity of the purified compounds with that of the total extract also suggests that the different isolated compounds probably act synergistically or at least additively.

### Anti-biofilm Activity of *S. officinalis* and Carnosol (Analogs)

The widespread mode of microbial growth in most ecological niches is as biofilm, and *C. albicans* is the most prevalent human fungal pathogen in both immunocompetent and immunocompromised individuals. It can cause both superficial and systemic infections, and the National Institutes of Health (NIH) estimates that 80% of human infections result from pathogenic biofilms (Harriott and Noverr, 2011). *Candida* biofilms are intrinsically resistant to conventional antifungal therapeutics; as a result, biofilm-associated infections pose a major clinical challenge (Gulati and Nobile, 2016). Previous studies on the anti-biofilm activities of *Salvia* species used its volatile oil. However, no study has thus far documented the antifungal biofilm properties of *S. officinalis* extracts, let alone isolated the *S. officinalis* compounds responsible for the *Candida* anti-biofilm activity.

### Safety of *S. officinalis* and Carnosol (Analogs)

According to Hamidpour et al. (2014), there are no reports of negative side effects associated with *S. officinalis*, and normal use of sage is not risky nor hazardous. In the Ames test on *Salmonella* it was found to have anti-mutagenic activity (Minnunni et al., 1992) and anticancer properties were found on several cell lines (Johnson, 2011). Not only *in vitro*, but several *in vivo* studies have suggested that daily oral administration of carnosol is well tolerated in animals. Methanol extracts from the leaves of *Salvia officinalis* (sage) could significantly inhibit serum triglyceride elevation in olive oil-fed mice (500 and 1,000 mg/kg, p.o.) (Ninomiya et al., 2004). Through bioassay-guided purification using the inhibitory activity against pancreatic lipase activity, four abietane-type diterpenes, including carnosic acid and carnosol, were isolated. Carnosic acid could inhibit triglyceride elevation in olive oil-fed mice at doses of 5–20 mg/kg (p.o.), while carnosol did not show any such effect, even at 200 mg/kg (Ninomiya et al., 2004). Johnson (2011) and his co-workers Johnson et al. (2010) found that oral carnosol (30 mg/kg 5 days weekly over a 28-day period) was well tolerated in mice; daily body weight measurements did not differ significantly between carnosol- and vehicle-treated animals. Also in Sprague-Dawley rats 1% carnosol in the diet for up to 2 weeks but had no observable effect on body weight (Singletary et al., 1996). Recently, the European Union (EU) approved rosemary extracts, standardized to diterpenes (e.g., carnosic acid and carnosol), and in the United States they were granted GRAS (Generally Recognized as Safe) status by the Food and Drug Administration (Petiwala and Johnson, 2015). All these data underscore the safety of oral carnosic acid and (some of) its analogs. A pharmacokinetic study of carnosol, rosmanol, and carnosic acid showed peak plasma levels of 27 μg/mL for carnosic acid (Wang et al., 2017), comparable to the BIC_50_ for our 12-methoxy-trans-carnosic acid, suggesting that therapeutically relevant antifungal concentrations could be reached with some of these compounds after oral administration.

A limitation of our study is that we have not exhaustively identified all antimicrobial components in the *S. officinalis* methanol extract. We have not pursued fractions with minor activity, and were unable to identify the bioactive compounds in some active chromatographic peaks due to the limited amount of material present. We found activity against non-*albicans Candida* species (*C. glabrata* and *C. parapsilosis*), and the activity pattern of the extracts in different solvents are dissimilar, suggesting that different compounds may underlie (at least in part) the differences in bioactivity. Further studies are needed to identify those compounds with antifungal activity against non-*albicans Candida* species.

In summary, we isolated carnosol and 12-methoxy-trans-carnosic acid from methanol extracts of *Salvia officinalis* and demonstrated that these are the two major compounds responsible for activity against *C. albicans* biofilm *in vitro*. Both compounds may have therapeutic potential by themselves or in combination with clinical antimicrobials. This will require more detailed evaluation in *in vivo Candida* biofilm models.

## Author Contributions

NK, SP, and WL conceived and designed the experiments. SP, NK, M-RY, and J-GL performed the experiments. SP, WL, M-RY, J-GL and Z-HJ analyzed the data. WL, Z-HJ contributed reagents, materials, and analysis tools; SP, WL, M-RY, Z-HJ, and HN contributed to the writing of the manuscript. All authors contributed to manuscript revision, read, and approved the submitted version.

### Conflict of Interest Statement

The authors declare that the research was conducted in the absence of any commercial or financial relationships that could be construed as a potential conflict of interest.

## Abbreviations

ATCC: American Type Culture Collection, Manassas, Virginia, USA
BIC_50_: Biofilm inhibitory concentration required to inhibit the growth of 50% of organisms
DMSO: Dimethyl sulfoxide
ESI: Electrospray ionization
HPLC: High performance liquid chromatography
LCMS: Liquid chromatography mass spectrometry
MDR: Multi-drug resistant
MHA: Mueller-Hinton agar
MIC: Minimum inhibitory concentration
NIH: National Institutes of Health
NMR: Nuclear magnetic resonance
OD: Optical density
RPMI: Roswell Park Memorial Institute
SD: Standard deviation
TFA: Trifluoro acetic acid
YPD: Yeast extract peptone dextrose.
