# Supplementary material for: Bioassay-Guided Isolation of Anti-Candida Biofilm Compounds From Methanol Extracts of the Aerial Parts of Salvia officinalis (Annaba, Algeria)
Source: Front Pharmacol. 2018 Dec 10;9:1418. doi: 10.3389/fphar.2018.01418 (PMC6295571; doi:10.3389/fphar.2018.01418)
Supplement: Supplementary file 1 [file Data_Sheet_1.docx]

Supplementary Material

# Bioassay-Guided Isolation of Anti-Candida Biofilm compounds From Methanol Extracts of the Aerial Parts of *Salvia officinalis* (Annaba, Algeria)

Neila Kerkoub^1¥*^, Sujogya Kumar Panda^2¥*^, Ming-Rong Yang^3^, Jing-Guang Lu^3^, Zhi-Hong Jiang^3^, Hichem Nasri^1^, Walter Luyten^2^

*** Correspondence**

Neila kerkoub (neilaker23@gmail.com ), Sujogya Kumar Panda (sujogyapanda@gmail.com)

# Supplementary Data (HRMS and NMR spectrum)

|  |
| --- |
|  |

**Figure 1.** HRMS spectrum and data of **WL-04**

| Formula | Score | *m/z* (Calc) | Diff(ppm) | Ion Formula | *m/z* |
| --- | --- | --- | --- | --- | --- |
| C_20_H_26_O_4_ | 100 | 331.1904 | -1.1 | C_20_H_26_O_4_ | 331.1908 |
| C_20_H_26_O_4_ | 57.38 | 329.1758 | -2.8 | C_20_H_26_O_4_ | 329.1768 |


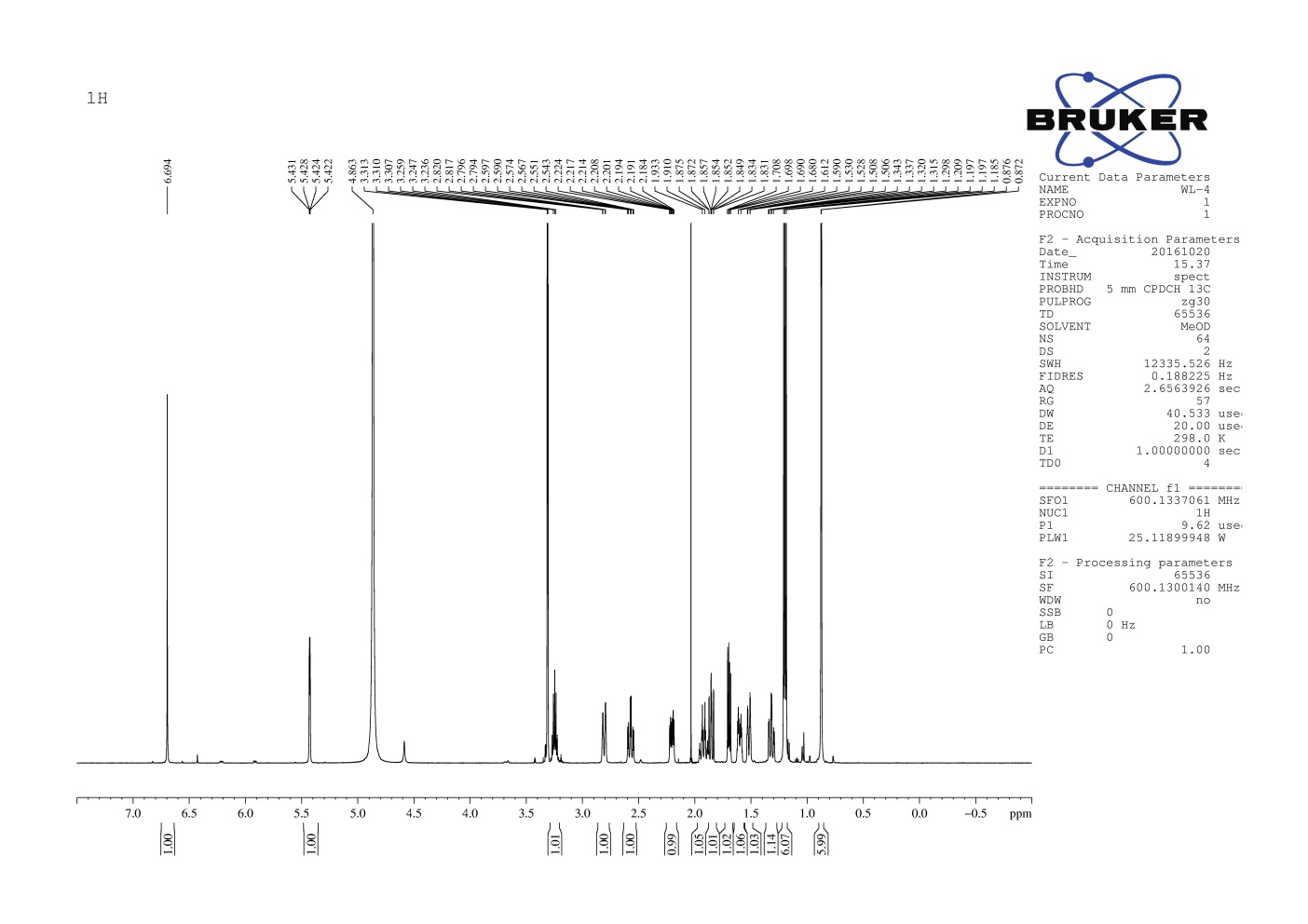


**Figure 2.** ^1^H-NMR spectrum of **WL-04**


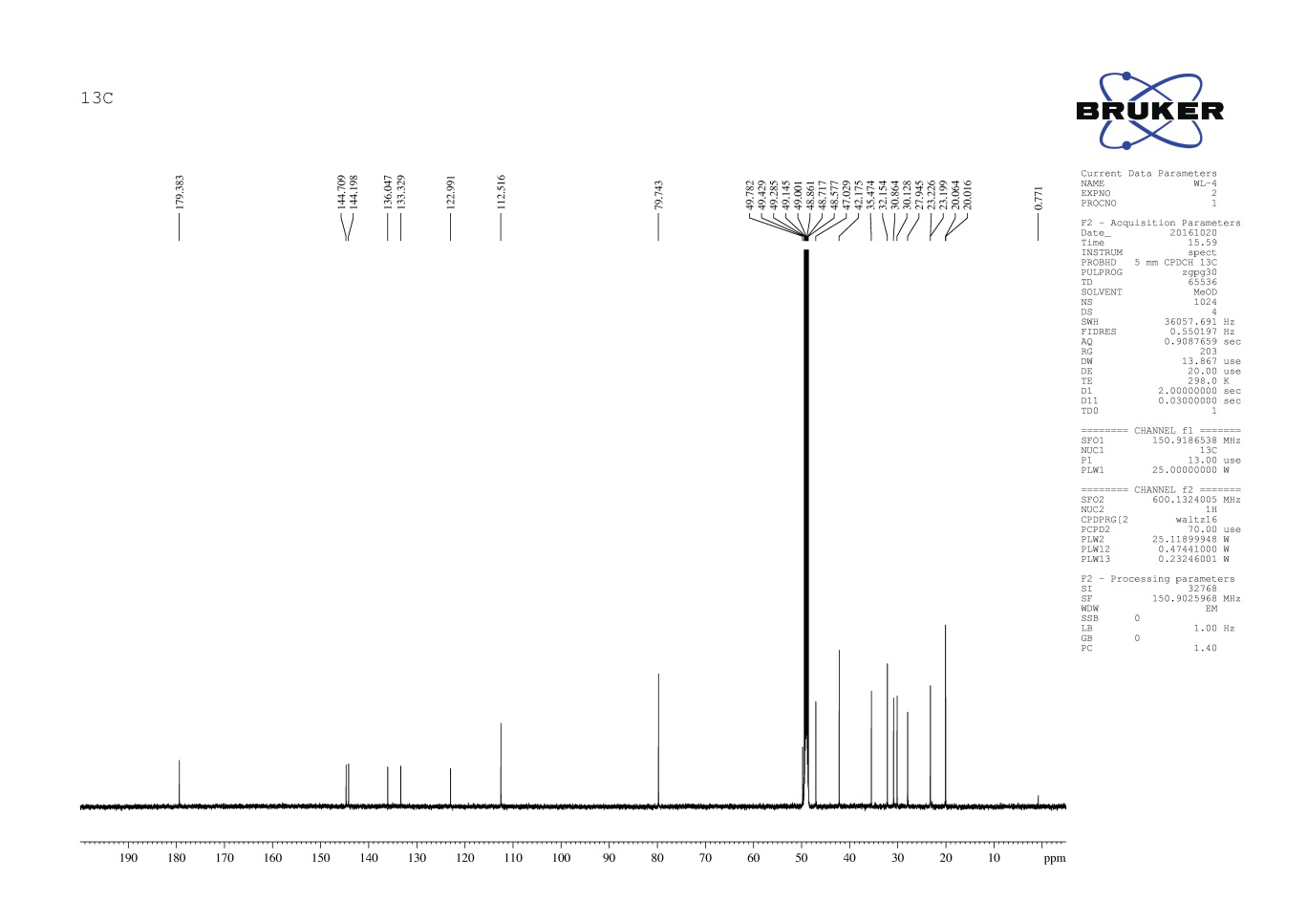


**Figure 3.** ^13^C-NMR spectrum of **WL-04**

|  |
| --- |
|  |

| Formula | Score | *m/z* (Calc) | Diff(ppm) | Ion Formula | *m/z* |
| --- | --- | --- | --- | --- | --- |
| C_21_H_30_O_4_ | 100 | 347.2217 | -0.2 | C_21_H_30_O_4_ | 347.2218 |
| C_21_H_30_O_4_ | 100 | 345.2071 | -3.4 | C_21_H_30_O_4_ | 345.2083 |

**Figure 4.** HRMS spectrum and data of **WL-12**


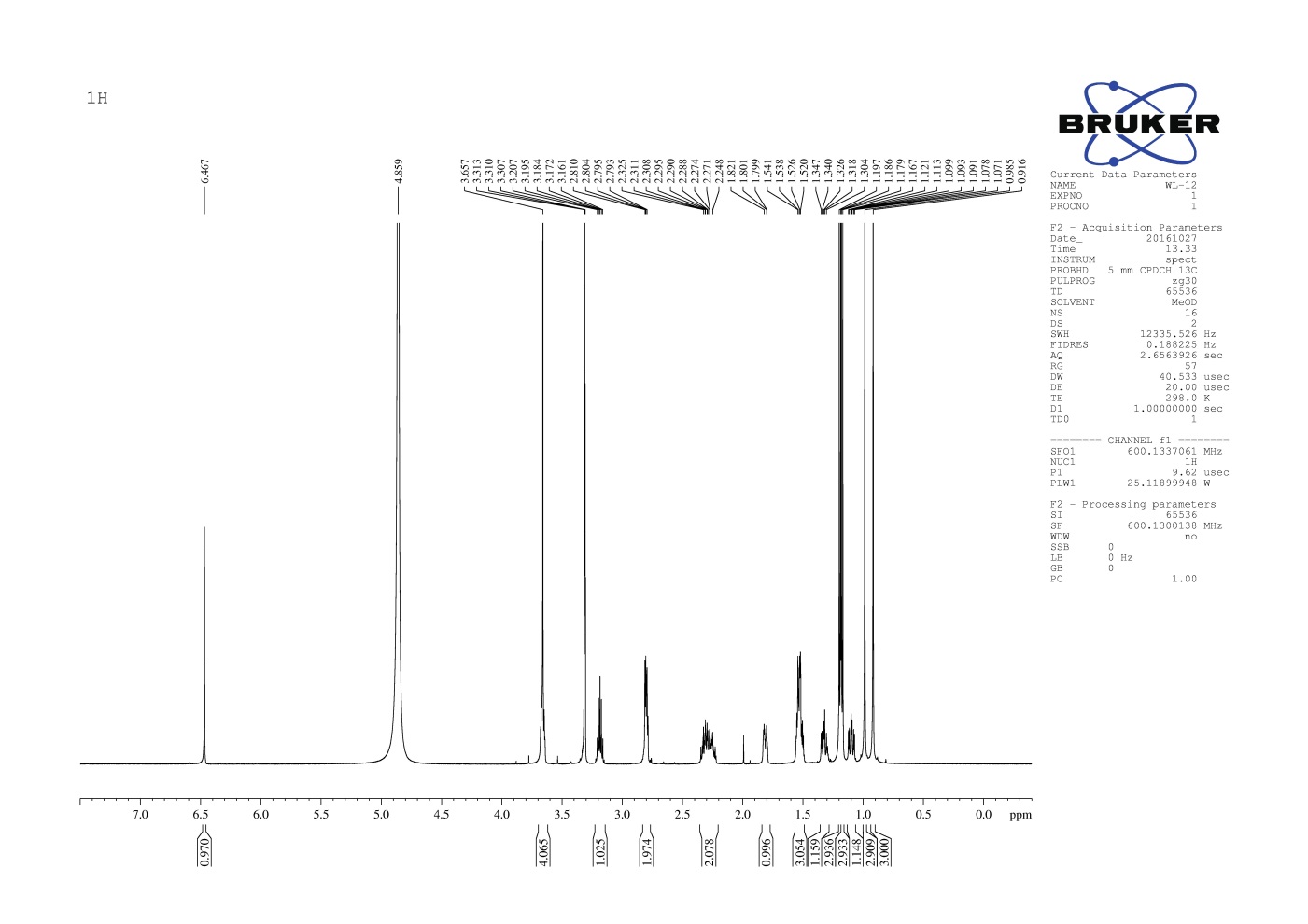


**Figure 5.** ^1^H-NMR spectrum of **WL-12**

***
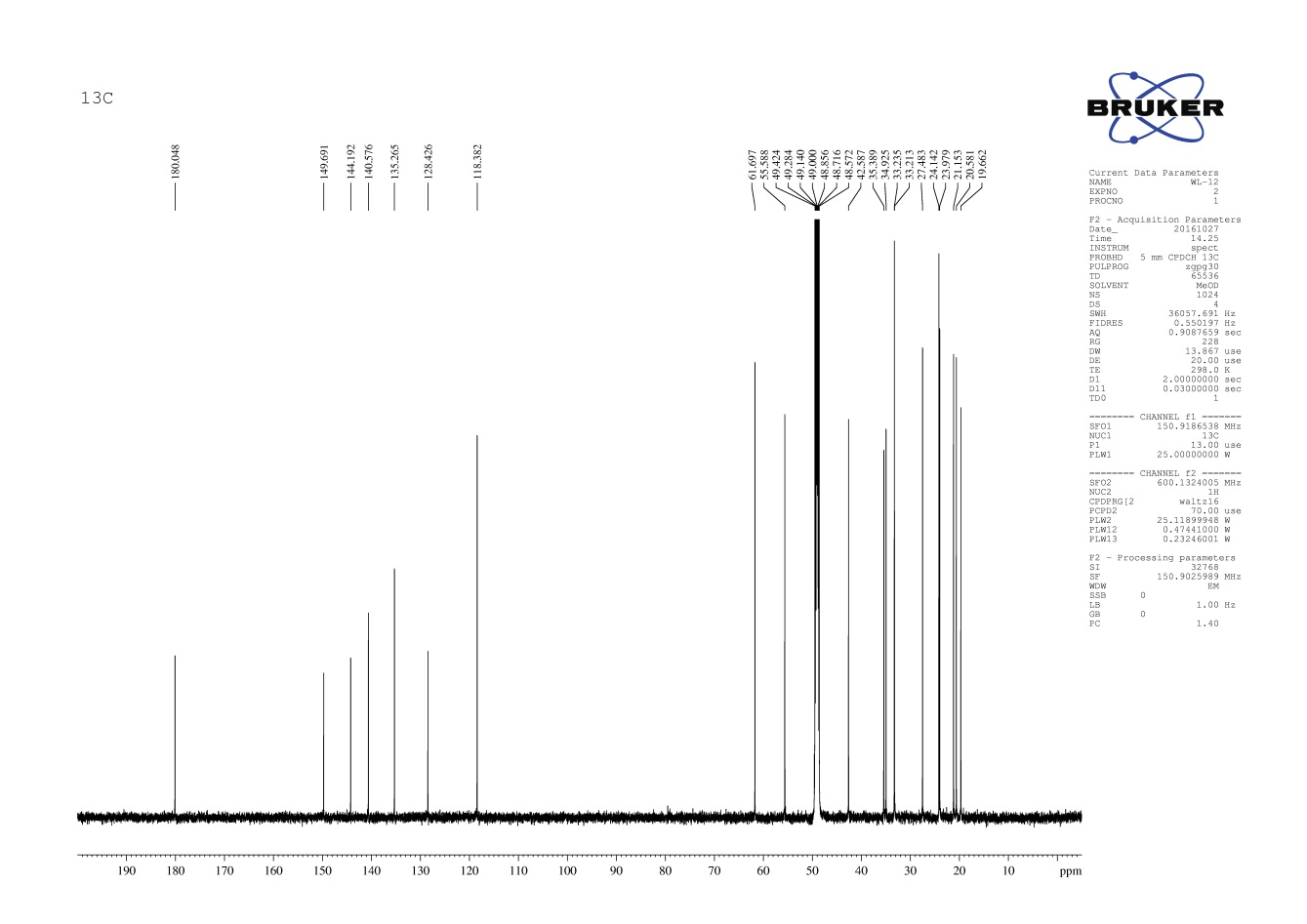
***

**Figure 6.** ^13^C-NMR spectrum of **WL-12**


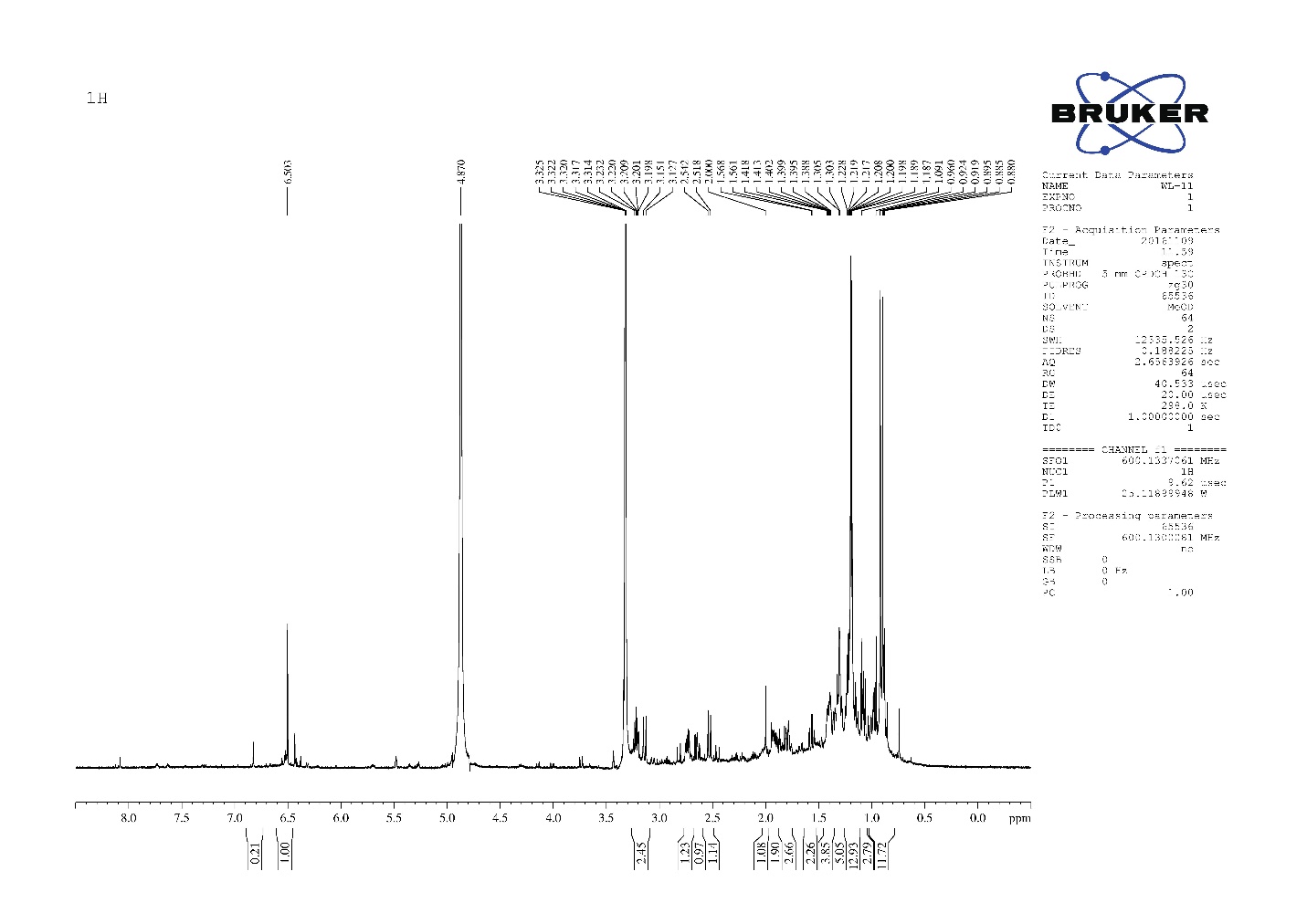


**Figure 7.** ^1^H-NMR spectrum of **WL-11**


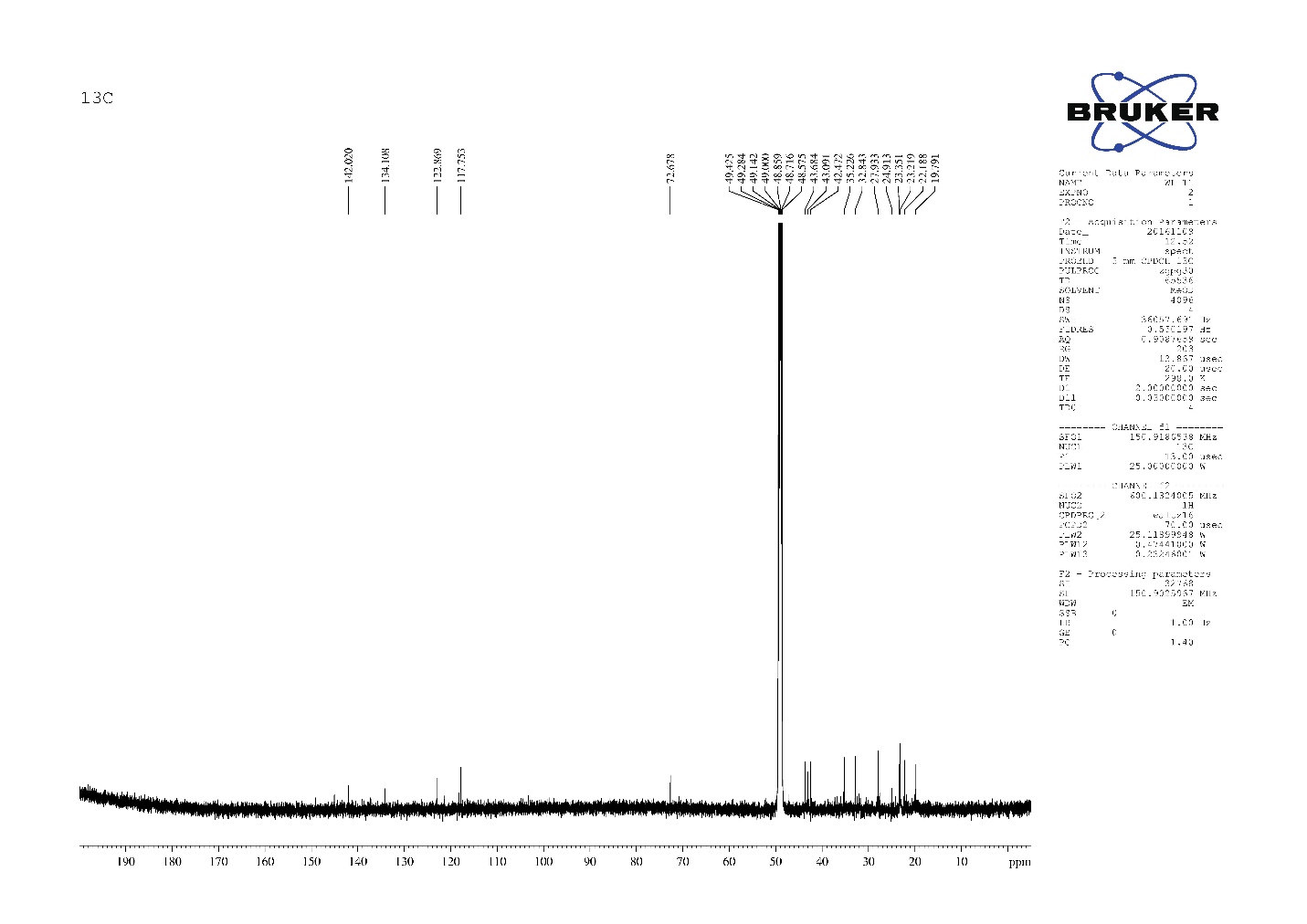


**Figure 8.** ^13^C-NMR spectrum of **WL-11**

**Figure 9.** HRMS spectrum and data of **WL-11;** [Formula: C_16_H_26_N_6_O ([M-H]^-^= 317.2095, -4.9ppm)
